# Phylogeographic patterns of the desert poplar in Northwest China shaped by both geology and climatic oscillations

**DOI:** 10.1186/s12862-018-1194-1

**Published:** 2018-05-25

**Authors:** Yan-Fei Zeng, Jian-Guo Zhang, Bawerjan Abuduhamiti, Wen-Ting Wang, Zhi-Qing Jia

**Affiliations:** 1 State Key Laboratory of Tree Genetics and Breeding, Research Institute of Forestry, Chinese Academy of Forestry, NO. 1 Dongxiaofu, Xiangshan road, Haidian district, Beijing, 100091 China; 2 Collaborative Innovation Center of Sustainable Forestry in Southern China, Nanjing Forestry University, No. 159 Longpan road, Nanjing, 210037 China; 3 Key Laboratory of Tree Breeding and Cultivation of the State Forestry Administration, Research Institute of Forestry, Chinese Academy of Forestry, No. 1 Dongxiaofu, Xiangshan road, Haidian district, Beijing, 100091 China; 4 Forest Research Institute of the Altai Region, No. 93 Jiefang South Road, Altai, Xinjiang, 836500 China; 5 School of Mathematics and Computer Science, Northwest University for Nationalities, No. 1 Northwest new village, Lanzhou, 730030 Gansu China; 6 Institute of Desertification Studies, Chinese Academy of Forestry, Xiangshan road, Haidian district, Beijing, 100091 China

**Keywords:** Arid northwest China, Climatic oscillations, Desert poplar, Ecological Niche Modeling, Phylogeography, Tianshan Mountains

## Abstract

**Background:**

The effects of historical geology and climatic events on the evolution of plants around the Qinghai-Tibetan Plateau region have been at the center of debate for years. To identify the influence of the uplift of the Tianshan Mountains and/or climatic oscillations on the evolution of plants in arid northwest China, we investigated the phylogeography of the Euphrates poplar (*Populus euphratica*) using chloroplast DNA (cpDNA) sequences and nuclear microsatellites, and estimated its historical distribution using Ecological Niche Modeling (ENM).

**Results:**

We found that the Euphrates poplar differed from another desert poplar, *P. pruinosa*, in both nuclear and chloroplast DNA. The low clonal diversity in both populations reflected the low regeneration rate by seed/seedlings in many locations. Both cpDNA and nuclear markers demonstrated a clear divergence between the Euphrates poplar populations from northern and southern Xinjiang regions. The divergence time was estimated to be early Pleistocene based on cpDNA, and late Pleistocene using an Approximate Bayesian Computation analysis based on microsatellites. Estimated gene flow was low between these two regions, and the limited gene flow occurred mainly via dispersal from eastern regions. ENM analysis supported a wider distribution of the Euphrates poplar at 3 Ma, but a more constricted distribution during both the glacial period and the interglacial period.

**Conclusions:**

These results indicate that the deformation of the Tianshan Mountains has impeded gene flow of the Euphrates poplar populations from northern and southern Xinjiang, and the distribution constriction due to climatic oscillations further accelerated the divergence of populations from these regions. To protect the desert poplars, more effort is needed to encourage seed germination and seedling establishment, and to conserve endemic gene resources in the northern Xinjiang region.

**Electronic supplementary material:**

The online version of this article (10.1186/s12862-018-1194-1) contains supplementary material, which is available to authorized users.

## Background

In eastern Asia, the effect of geological dynamics, e.g., the uplift of the Qinghai-Tibetan Plateau (QTP) and Quaternary climatic oscillations, on species diversification has been at the center of debate for several years [1–4]. A lot of phylogeography studies have focused on the speciation and population divergence of organisms in the Hengduan Mountains and adjacent regions in southwest China, which harbors one of the world’s major plant diversity hotspots [3]. Studies have found that orogeny creates conditions favoring rapid in situ speciation of resident lineages [4, 5], and the lowland refugia for plants throughout climatic oscillations in the Quaternary further maintain high biodiversity in these regions [2]. Recently, a growing number of studies have begun to pay attention to plants near the northern edge of the QTP in the arid northwest area of China [6]. However, the possible roles of geology and climatic oscillations in facilitating the population divergence and species diversification of plants in arid northwest China are still poorly understood.

Northwest China is located in central Asia and is arid or semi-arid, with a cold and dry continental climate. The progressive extension of the uplift of the QTP was associated with the orogeny of the Tianshan Mountains, which achieved a significant elevation during the Miocene [3, 7]. The rise of the Tianshan Mountains and the Higher Himalayas massively altered air circulation. Meanwhile, a worldwide cooling has occurred since the Middle Miocene climate optimum. These together have caused the progressive aridification of central Asia [8]; see [3] for more references. Other factors, such as changes in the global ice volume and the final disappearance of the Tethys Sea in Asia (late Miocene and early Pliocene), and the intensification of the East Asian summer and winter monsoons (EASM and EAWM) also contributed to the aridification of central Asia [9, 10]. Currently, the deserts of northwest China, including the four largest deserts (Taklamakan, Guerbantonggute, Badain Jaran, and Tengger), together comprise the world’s largest mid-latitude, temperate, continental interior desert region [11, 12].

The flora of arid northwest China constitutes mainly Tethys coastal xerophytes [13]. The Tianshan Mountains, running roughly E-W for about 2500 km, are located between the Tarim and Dzungarian basins and separate the Taklamakan and Guerbantonggute deserts, and therefore may have profoundly affected the genetic structure and distribution patterns of desert plants in northwest China. For example, these mountains are suggested to have triggered the separation of different *Aconitum nemoru* lineages [14]. Although the major Quaternary glaciations were absent in arid northwest China, the evolution of the biota there have been impacted by significant climatic oscillations [6]. The further development of the East Asian monsoon system, especially the increasing variability and strengthening of the EAWM, may have influenced the onset of major Northern Hemisphere glaciations after 2.6 Mya, and accelerated the further aridification of central Asia during the Pliocene and Pleistocene [10]. Previous phylogeographical studies have found that desert expansion caused habitat fragmentation and aridification during the last glacial maximum (LGM), which promoted the diversification and speciation of desert plants [15], see [6] for more references. Although a number of recent studies have examined the effects of a particular process in a limited geographic region [6], few have considered the relative importance of geological and climatic dynamics over a wide area. Related studies may facilitate our understanding the roles of geology and climatic oscillations in driving population divergence of plants in this arid zone, and comparing them with those in the diversity hotspots regions in southwest China, which would give an insight into the emergence and maintenance of biodiversity.

Two closely related desert poplars, the Euphrates poplar (*Populus euphratica* Olivier) and *P. pruinosa* Schrenk, are the only tree species that have established in the world’s largest shifting-sand desert, the Taklimakan Desert [16]. *P. euphratica* is naturally distributed in western China and adjacent Middle-Eastern countries. More than 61% of *P. euphratica* forests occur in China, of which about 91.1% are located in Xinjiang province [16]. *P. pruinosa* has a more restricted distribution than *P. euphratica*, occurring in alluvial oasis communities in northwest China, Kazakstan, Tajikistan, Turkmenistan, and Uzbekistan [17]. Recent studies suggest that gene flow and Pleistocene climate oscillations might have triggered this speciation [17, 18]. Populations of *P. euphratica* and *P. pruinosa* are endangered as a consequence of intensive water use and damming in northwest China [19, 20]. As a typical species of Tethys, the Euphrates poplar might have existed before the uplift of the Tianshan Mountains, and therefore it is a suitable species in which to study the influence of geological and climatic dynamics on species divergence in arid northwest China.

In this study, we used both chloroplast and nuclear markers to explore the phylogeographical pattern of these two desert poplars in northwest China, with a particular focus on the widespread Euphrates poplar, and used Ecological Niche Modeling (ENM) to estimate their potential historical distributions, and finally identify how the uplift of the Tianshan Mountains and Quaternary climatic oscillations influenced the current population structure of desert plants*.* More specifically, the aim was to characterize: (1) the pattern of genetic diversity of the two desert poplars in northwest China; (2) the divergence and gene flow between Euphrates poplar populations to the north and the south of the Tianshan Mountains; and (3) the historical population demography of Euphrates poplar in response to climatic oscillations.

## Methods

### Sampling and DNA extraction

Leaf samples from 552 *P. euphratica* individuals were collected from 33 natural populations (12 from northern Xinjiang, 14 from southern Xinjiang, and seven from Qinghai province, Gansu province, and Inner Mongolia province (the ‘QGM’ region)), which covers the whole range of this species in northwest China (See Additional file 1: Figure S1 and Table S1). Leaf samples of 102 *P. pruinosa* individuals were collected from five natural populations in Xinjiang province, of which four co-occurred with *P. euphratica*. All sampled individuals were at least 20 m apart from each other in any given population. Leaf tissues were dried with silica gel and taken to the laboratory. Total genomic DNA was extracted from 25 mg of leaf tissue from each tree and purified using a Plant Genomic DNA Extraction Kit (Tiangen, Beijing, China). In addition, DNA samples from three *P. ilicifolia* supplied by the Royal Botanic Gardens (http://www.kew.org/) were used for chloroplast DNA (cpDNA) sequence analysis. All collection of specimens used in our study complied with related institutional, national, and international guidelines.

### Microsatellite marker procedure

The samples were screened for variation at 17 nuclear microsatellite loci that were either supplied by the International Populus Genome Consortium from genome sequence of *P. trichocarpa* [21] or developed for *P. euphratica* [22]; see Additional file 1: Table S2 for primer details. We used an economic method suggested by Schuelke [23] for fluorescent dye labeling of the polymerase chain reaction (PCR) fragments. This was performed using a three-primer protocol including unlabeled M13-tagged forward and unlabeled/untagged reverse primers for each marker and a third ‘universal’ M13-primer labelled with one of the fluorescent dyes, 6-FAM, HEX, or TAMRA (Sangon, Shanghai, China). PCR amplifications were performed according to Schuelke [23]. Microsatellite genotypes were resolved on an ABI 3130XL automated sequencer (Life Technologies, Foster City, CA, USA), for which allele sizing was performed using the GENEMAPPER software version 4.0 (Life Technologies), with the Liz 500 (Life Technologies) as an internal standard.

### Chloroplast sequence procedure

The chloroplast *trn*K region was amplified using the primers *trn*K-1682F (5′- GGGTTGCCCGGGACTCGAAC-3′) and *trn*K-4292R (5′- TGGGTTGCTAACTCAATGG-3′), which were improved from the Demesure et al. [24] original, according to the *P. trichocarpa* chloroplast complete genome (GenBank accession numbers NC_009143) [21]. A PCR was performed in a 25-μL volume, following the methods described by Zeng et al. [25]. Sequencing reactions were performed with the PCR primers and two more in-between primers, matK-2332F (5′- ACTAATGGGATGTCCTACTG-3′) and matK-3741R (5′- GATTTCTAGTCACCTATTAC-3′), to cover the whole PCR segment, using an ABI Prism BigdyeTM terminator cycle sequencing ready reaction kit (Life Technologies). The reaction mixtures were analyzed on an ABI 3130xl automated sequencer (Life Technologies).

## Microsatellite data analysis

### Genetic diversity and differentiation

Clonal reproduction is common in *P. euphratica and P. pruinosa* [26]. To identify the clonal lineage of each population, multilocus genotype assignments were conducted using the GenoType software [27], by calculating a pairwise distance matrix under an infinite allele mutation model. A threshold of two was used to define the same clonal lineage after drawing a frequency distribution of the values of all pairwise comparisons (See Additional file 1: Figure S2).

Clonal diversity was estimated as:

$$ R=\frac{G-1}{N-1} $$

with *G* representing the number of multilocus genotypes (MLGs) or multilocus lineages (when taking into account possible somatic mutations or scoring errors) discriminated in the sample and *N* representing the number of sampled ramets.

After the identification of ramets belonging to the same genets, replicates were removed from the data set to perform a subsequent analysis. The number of alleles, allele frequencies, and observed and expected heterozygosity (*H*_O_ and *H*_E_), were calculated for each microsatellite locus using the FSTAT 2.9.3 program [28]. With this program, genetic diversity in terms of total allele numbers (*A*), *H*_O_, *H*_E_, and the inbreeding coefficient (*F*_IS_) were calculated for each population across all microsatellite loci. We used a permutation procedure (1000 permutations) to test whether a particular estimate of the overall *F*_IS_ was significantly different from 0. For *P. euphratica*, the regional genetic diversity (northern Xinjiang, southern Xinjiang, and QGM regions) was also calculated using values of *H*_O_, *H*_S_ (average of *H*_E_ for subpopulations), and *F*_IS_. Allelic richness (*A*_R_) and private allelic richness (*P*_A_) for each of the three regions were calculated by standardization for 25 individuals, using the hierarchical sampling method as executed in hp_rare 1.0 [29]. For both species, population differentiation among populations and among regions were estimated by *F*_ST_ with FSTAT [28].

### Bayesian cluster analysis

We used a Bayesian model-based clustering method implemented in the program STRUCTURE version 2.3 [30–32] to first identify *P. euphratica* or *P. pruinosa* ancestry for all individuals, and then to detect the population structure of *P. euphratica* for individuals only with *P. euphratica* morphology. Based on the LOCPRIOR model described by Hubisz et al. [33], the program was run based on genet MLG data, with a correlated allele frequency model (*F*-model) and an admixed origin of populations. After an initial test, where we varied the burn-in and run length, the burn-in was set to 500,000 with 1000,000 additional cycles. For both data sets, 20 replicate runs were conducted for each value of K from one to ten. The final posterior probability of *K*, Pr(X|*K*), and *ΔK*, where the modal value of the distribution is located at the real *K* [34], were both used as an indication of the most likely number of clusters. For graphic visualization of the STRUCTURE results, we used DISTRUCT [35].

### Population history inference

To identify the possible population history of *P. euphratica*, five alternative scenarios for three population groups, northern and southern Xinjiang, and the QGM region, were tested using the Approximate Bayesian Computer procedure [36] as performed in DIYABC v.2.1.0 [37]. Scenarios 1, 2, and 3 tested a ‘colonization’ event from the QGM region, and northern and southern Xinjiang, respectively. Scenario 4 tested a split of the three groups at the same time. In Scenario 5, populations from the QGM region were created by an admixture of two separated gene pools from northern and southern Xinjiang. We set a varied ancestry population size before the colonization or split of the populations (See Additional file 1: Figure S3).

One million simulations were run for each scenario. Prior parameter distributions for population sizes and time frames (measured in generations) were set as following: uniform (100; 100,000) for both current and ancestral effective population sizes, uniform (10; 10,000) for divergence times t1, uniform (10; 50,000) for t2 (with t2 > t1), and uniform (0.001; 0.999) for the admixture rate. *P. euphratica* begins to reproduce at the age of 8–10 years and reaches flourishing period at the age of 15–30 years [38]; we thus considered 20 years to represent a reasonable generation time.

### Historical and contemporary gene flow

To estimate the gene flow pattern among regional population groups of *P. euphratica* (northern and southern Xinjiang, and the QGM region), we used the coalescent approach implemented in Migrate-n version 3.6 [39]. This program calculates maximum likelihood (ML) estimates for both effective population size (*θ* = 4Neμ, where μ = mutation rate) and historical migration rates (M = m/μ, where m = migration rate) between pairs of population groups [40]. Analyses were run for five replicates using a Brownian motion mutation model, with constant mutation rates and starting parameters based on *F*_ST_ calculations. For each replicate, we used 10 short chains (10,000 trees) and three long chains (200,000) with 100,000 trees discarded as an initial ‘burn-in’ and a static heating scheme at four temperatures (1, 1.5, 3, and 1000,000). To avoid the confounding effects of the differences in sample size on the estimate of gene flow, the software randomly picked 40 individuals from each group.

To estimate contemporary gene flow (m) between the above three regional population groups of *P. euphratica*, we used the software BayesAss 3.0 [41], which carries assignment tests in a Bayesian framework and a Markov coupled Markov chain (MCMC). After a burn-in of 2 × 10^6^, we ran the analyses for 10^7^ iterations, with a sampling frequency of 10^3^. Delta values for the migration (m), allele frequencies (a) and inbreeding (f) switching proposals were adjusted so that the accepted numbers of changes were 30–50% of the total number of iterations. The delta values for a, m, and f were 0.2, 0.1, and 0.2, respectively. We performed 20 runs (each with a different seed) and selected the one with the lowest deviance for further analyses [42].

### CpDNA sequence analyses

Sequences of *trn*K were aligned using ClustalX ver. 1.81 (Thompson et al.*,* 1997) with subsequent manual adjustments. A matrix of *trn*K sequences was constructed for the 286 trees that we examined, and different cpDNA sequences were identified as haplotypes. The sequences for those haplotypes have been deposited in GenBank under accession numbers KY002202–KY002230. Average chloroplast gene diversity within populations (*H*_S_) and total gene diversity (*H*_T_) were calculated using the program ARLEQUIN version 3.1 [43] for each species and each region of *P. euphratica*, respectively. The measure of population differentiation, *G*_ST,_ was estimated as (*H*_T_-*H*_S_)/*H*_T_. Relationships between haplotypes were examined via a haplotype network, which was constructed using the computer program Network version 4.5.1.6 [44]. Phylogenetic relationships and divergence times among haplotypes were estimated using Bayesian inference methods implemented in BEAST v.1.8.2 [45], using a sample with each of *P. alba* and *P. laurifolia* as outgroups and the substitution rate of the chloroplast sequence estimated in *P. balsamifera* and *P. trichocarpa* (μ = 3.46 × 10^− 10^ s s^− 1^ y^− 1^; [46]). The General Time Reversible (GTR) nucleotide substitution model was used in the program. A normal prior probability distribution was used to accommodate the uncertainty of the prior knowledge. We sampled all parameters once every 1000 steps from 10^7^ MCMC steps, with the first 25% of samples discarded as the burn-in. The consensus trees were generated by TreeAnnotator v.1.8.2 [45].

### Ecological niche modeling

Species distribution models for *P. euphratica* and *P. pruinosa* were generated using MAXENT 3.3.3e [47], to predict species occurrence under present-day, LGM, last interglacial (LIG), and three million years ago (Pliocene) conditions. In addition to our sample locations, the distribution records for the Euphrates poplar were sourced from the Chinese Virtual Herbarium (http://www.cvh.ac.cn/), Global Biodiversity Information Facility (http://www.gbif.org/), and previously published papers [17, 38, 48]. The locations where species occurred that were within 24 km of one another (12 arc-min) were removed to reduce the effects of spatial autocorrelation in climate variables. The ecological layers for the current climate were obtained from the WorldClim database (http://www.worldclim.org/version1) [49]. For the LGM prediction, data were taken from general circulation model simulations using the Community Climate System Model (CCSM) [50] and the Model for Interdisciplinary Research on Climate (MIROC) [51]. The LIG distributions for these two species were predicted using the climatic model developed by Otto-Bliesner et al. [52]. For the Pliocene prediction, data provided by Lunt et al. [53] were used.

For *P. euphratica*, the following five uncorrelated and biologically significant bioclimatic variables were selected as predictors: (1) mean diurnal range (mean of monthly maximum temperature - minimum temperature), (2) minimum temperature of the coldest month, (3) mean temperature of the wettest quarter, (4) precipitation of the driest quarter, and (5) precipitation of the warmest quarter. For *P. pruinosa*, (1) annual mean temperature, (2) temperature annual range (maximum temperature of warmest month - minimum temperature of coldest month), (3) precipitation of wettest month, (4) precipitation of driest month, and (5) precipitation of coldest quarter were used. After a model testing with 25% of the data, model validation was then performed 20 independent replicates using default settings. For each run, the area under the receiver operating characteristic curve was calculated as an indicator of the accuracy of model prediction. The ENM was first analyzed based on worldwide records of the Euphrates poplar, and then analyzed based on records in China only to specify the suitability of its estimation in China.

## Results

### Microsatellite variation

The 17 microsatellite loci yielded a total of 228 alleles (2–28 per locus) from our sample of 673 individuals (See Additional file 1: Table S3), of which 207 and 152 alleles were detected in *P. euphratica* and *P. pruinosa*, respectively. Following our analyses based on these microsatellite loci, 54 distinct MLGs were detected from the 122 samples of *P. pruinosa*, and 203 MLGs were detected from 551 samples of *P. euphratica*, with the clonal diversity (R) ranging from 0.077–0.821 within populations of *P. pruinosa*, and 0–0.947 within populations of *P. euphratica* (Table 1 and Additional file 1: Figure S1).

**Table 1:** Genetic diversity of each population of *Populus pruinosa* and *P. euphratica*

	Nuclear Microsatellites							Chloroplast sequence		
Code	*N*_s_	*MLGs*	R	A	*H*_O_	*H*_E_	*F*_IS_	*N*_c_	*N*_h_	Hap_div
*P. pruinosa*	*122*	*54*	*0.438*	*152*	*0.484*	*0.560*	*0.112* ^*^	*58*	*8*	*0.730*
MYp	15	8	0.500	67	0.493	0.590	0.175	9	3	0.667
BCp	14	2	0.077	35	0.441	0.480	0.118	4	2	0.500
Alp	16	8	0.467	68	0.477	0.520	0.089	12	3	0.530
GMp	40	33	0.821	135	0.539	0.595	0.094	26	5	0.668
YLp	17	3	0.125	46	0.471	0.549	0.172	7	2	0.476
*P. euphratica*	*551*	*203*	*0.368*	*207*	*0.538*	*0.558*	*0.046*	*225*	*20*	*0.705*
MYe	16	8	0.467	77	0.596	0.563	−0.062	10	1	0.000
BCe	14	8	0.538	79	0.581	0.588	0.013	11	4	0.691
Ale	17	8	0.438	72	0.412	0.541	0.251^a^	9	3	0.417
MF	16	10	0.600	74	0.505	0.539	0.065	11	3	0.473
PSh	16	3	0.133	49	0.569	0.541	−0.064	9	2	0.556
QM	18	1	0.000	24	*–*	*–*	*–*	2	1	0.000
YPH	20	9	0.421	73	0.473	0.504	0.065	9	2	0.222
RQ	16	10	0.600	70	0.444	0.512	0.140	11	1	0.000
ShY	17	10	0.588	99	0.547	0.585	0.068	10	2	0.200
KEL	14	12	0.846	65	0.477	0.492	0.032	10	3	0.378
WL	25	13	0.500	75	0.511	0.511	0.000	6	1	0.000
LT	7	1	0.000	26		–	–	1	1	–
YLe	13	6	0.417	69	0.549	0.579	0.057	7	4	0.810
ChJ	16	10	0.600	76	0.527	0.576	0.088	11	4	0.491
KLM	21	4	0.150	62	0.559	0.599	0.077	5	3	0.800
MGCa	15	2	0.071	51	0.549	0.612	0.125	4	2	0.500
MGCb	17	1	0.000							
BEJa	20	11	0.526	84	0.512	0.553	0.077	11	4	0.600
BEJb	20	7	0.316	58	0.626	0.575	−0.096	2	2	1.000
HBH	16	4	0.200	52	0.618	0.545	−0.161	4	2	0.500
BLK	3	1	0.000	70	0.588	0.593	0.099			
BLST	5	1	0.000					12	4	0.652
BLBQ	9	4	0.735							
QHX	3	1	0.000	25	–	–	–	3	1	0.000
MLX	20	1	0.000	24	–	–	–	12	1	0.000
YWX	24	15	0.625	96	0.539	0.582	0.099	12	4	0.803
TLH1	16	1	0.000	41	0.559	0.598	0.095	8	4	0.750
TLH2	35	1	0.000							
AKS	11	5	0.400	54	0.612	0.527	−0.185	5	2	0.400
JQ	23	1	0.000	23	–	–	–	2	1	0.000
DH	28	1	0.000	24	–	–	–	7	1	0.000
JT	20	19	0.947	83	0.489	0.509	0.040	7	3	0.762
EQN	20	14	0.684	94	0.538	0.584	0.082	14	4	0.648
Total	673	257	–	228	–	–	–	268	25	–

Number of samples genotyped (*N*s), number of multilocus genotypes (*MLGs*) identified, clonal diversity (R) and total number of alleles on the entire sample (A), expected heterozygosity (*H*_e_) and deviation from Hardy–Weinberg equilibrium (*F*_IS_, ^a^significant after a 1000 permutation test) with the 17 nuclear microsatellites. Number of samples sequenced (*N*_c_), number of haplotypes (*N*_c_) and haplotype diversity (Hap_div) with the chloroplast *trn*K sequences

After removing replicates belonging to identical MLGs, the overall gene diversity (*H*_T_) was similar for *P. euphratica* (0.594) and *P. pruinosa* (0.565). The mean values of *H*_O_ and *H*_E_ respectively across populations were 0.538 and 0.558 for *P. euphratica*, and 0.484 and 0.560 for *P. pruinosa*. After using a sequential Bonferroni correction, only one *P. euphratica* population that coexisted with *P. pruinosa* individuals, ALe, had a fixation index (*F*_IS_) that significantly deviated from zero, suggesting that all populations except ALe are under a Hardy–Weinberg equilibrium. No *F*_IS_ significantly deviated from zero for each population of *P. pruinosa*; but there was a significant deviation for the species as a whole, possibly reflecting the existence of a Wahlund effect (Table 1).

When all *P. euphratica* populations were divided into three regions, the regional genetic diversity, as *A*_R_, *P*_A_, *H*_O_, and *H*_S_, for northern Xinjiang was relatively high compared with southern Xinjiang and the QGM region; while the *F*_IS_ value was slightly higher for southern Xinjiang than the other two regions (Table 2).

**Table 2:** Comparison of genetic diversity and differentiation among three geographical populations of *Populus euphratica*

Group	Microsatellite						Chloroplast			
	*A*_R_	*P*_A_	*H*_O_	*H*_S_	*F*_IS_	*F*_ST_	*N*_H_	*H*_T_	*H*_S_	*G*_ST_
Northern Xinjiang	7.25	1.39	0.561	0.583	0.038	0.047	13	0.828	0.594	0.283
Southern Xinjiang	6.76	0.98	0.506	0.536	0.057	0.020	9	0.344	0.312	0.093
QGM region	6.98	0.71	0.525	0.542	0.032	0.028	7	0.646	0.423	0.345

*A*_R_, Allelic richness; *P*_A_, private allelic richness; *H*_O_, observed heterozygosity; *H*_S_, gene diversity; *F*_IS_, fixation index; *F*_ST_, among population differentiation. The analyses of *A*_R_ and *P*_A_ are based on 50 gene copies

### Population differentiation and structure

The interspecific genetic differentiation based on the Weir and Cockerham [54] estimator was moderate and significant across all microsatellite loci (θ = 0.328 ± 0.073, *p* < 0.01). Differentiation among populations was low within both species, but was relatively higher in *P. euphratica* (θ = 0.062 ± 0.005, p < 0.01) than in *P. pruinosa* (θ = 0.009 ± 0.007, *p* > 0.05). For *P. euphratica*, the *F*_ST_ values among populations within northern Xinjiang (0.047) were higher than those within both southern Xinjiang (0.020) and the QGM region (0.028, Table 2).

**Fig. 1:**
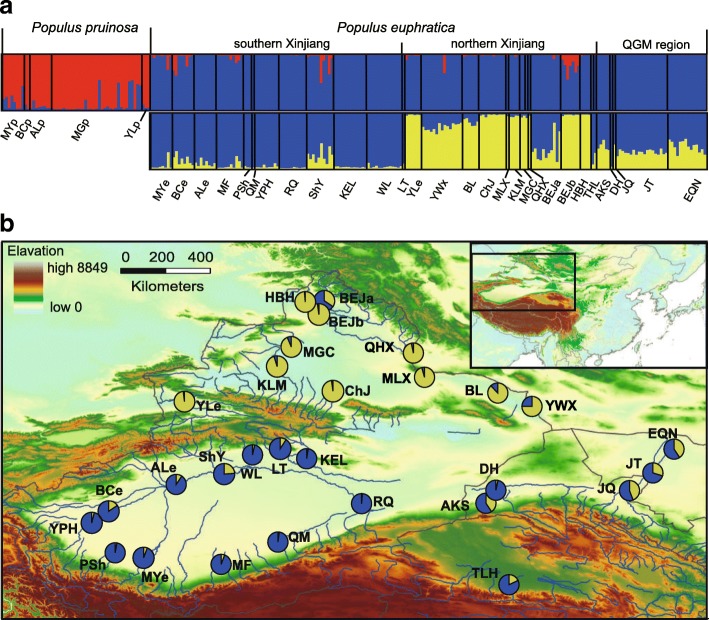
Bayesian estimation of the proportion of genetic clusters for each multilocus genotype (MLG) and population using STRUCTURE software. Analyses were conducted based on 17 microsatellite loci. (**a**) Proportion of genetic clusters at *K* = 2 for 257 MLGs of *Populus euphratica* and *P. pruinosa*, and 203 MLGs of *P. euphratica*. The smallest vertical bar represents one MLG. The assignment proportion of each MLG into the population clusters is shown along the y-axis. (**b**) Geographical distribution of the two genetic clusters and composition of the genetic cluster in each population of *P. euphratica*. The map was created using the ArcMap package in ArcGIS ver. 9.2 (http://www.esri.com/software/arcgis)

The STRUCTURE output for all MLGs showed that both likelihood (Ln P(D)) and *ΔK* supported the existence of two clusters (See Additional file 1: Figure S4a and b), which corresponded well with our morphological assignment to *P. euphratica* and *P. pruinosa* (Fig. 1a). The *P. euphratica* populations could be further classified into two clusters (See Additional file 1: Figure S4c and d), which corresponded approximately to populations from southern and northern Xinjiang, with the population BEJa from northern Xinjiang being an exception (Fig. 1b). Almost all populations from the QGM region were a mixture between the two Xinjiang clusters.

### Population history and gene flow

A comparison of the posterior probabilities (PPs) of the five scenarios using local linear regression indicated that Scenario 5 was the most likely (PP = 0.97, 95% CIs: 0.95–0.98; See Additional file 1: Figure S3). For Scenario 5, the median values of t1 and t2 were 336 generations (95% CIs: 65–1610) and 18,500 generations (95% CIs: 8100–32,800), respectively (Table 3). The ra composition of the southern Xinjiang group was 34.2%, while the median values of the effective population sizes of northern Xinjiang, southern Xinjiang, the QGM region, and ancestral population were 84,000, 80,400, 17,700, and 2780, respectively.

**Table 3:** Demographic approximate Bayesian computation models for *Populus euphratica* at scenario 5

Parameter	median	5%	95%
N1	8.40E + 04	5.53E + 04	9.82E + 04
N2	8.04E + 04	5.08E + 04	9.73E + 04
N3	1.77E + 04	4.39E + 03	6.77E + 04
Na	2.78E + 03	4.99E + 02	1.15E + 04
t1	3.36E + 02	6.50E + 01	1.61E + 03
t2	1.85E + 04	8.10E + 03	3.28E + 04
ra	3.42E-01	7.22E-02	7.75E-01
Вμmic_1	5.26E-05	3.20E-05	8.39E-05
pmic_1	6.58E-01	4.37E-01	8.71E-01

N1, N2, and N3, current effective population size of gene pools from the northern Xinjiang, southern Xinjiang, and the QGM region, respectively; Na, ancestral effective population size; t1–t2, estimated times of the different events depicted in Additional file 1: Figure S3 (in generations); ra, admixture rate of gene pools from southern Xinjiang; Вμmic_1, estimated microsatellite mutation rate; pmic_1, the parameter of the geometric distribution

**Fig. 2:**
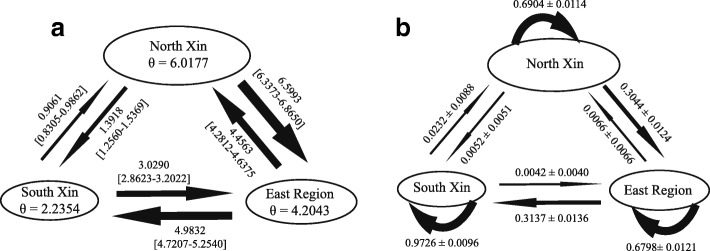
Gene flow among *Populus euphratica* populations from three regions. (**a**) Effective population size for each of the three regions and the historical gene flow among populations estimated by Migrate; (**b**) contemporary gene flow among the three regions estimated by BayesAss

Estimates of gene flow generated using Migrate were low between northern and southern Xinjiang, *4Nm*_north → south_ = 0.91 and *4Nm*_south → north_ = 1.39. The highest level of migration occurred from northern Xinjiang to the QGM region (*4Nm*_north → QGM_ = 6.60, 95% CIs: 6.34-6.86), followed by the QGM region to southern Xinjiang (*4Nm*_QGM → south_ = 4.98, 95% CIs: 4.72–5.25), resulting in a relatively higher gene flow from northern Xinjiang to southern Xinjiang via the QGM region than the reverse (*4Nm*_south → QGM_ = 3.03, 95% CIs: 2.86-3.20 and *4Nm*_QGM → north_ = 4.46, 95% CIs: 4.28–4.64; Fig. 2a).

The BayesAss analysis showed that the mean contemporary gene flow (m) among pairs of the three groups ranged from 0.0042 to 0.3137 (Fig. 2b). The highest level of migration occurred from the QGM region to southern Xinjiang (*m*_QGM → south_ = 0.3137 ± 0.0136), followed by migration from northern Xinjiang to the QGM region (*m*_north → QGM_ = 0.3044 ± 0.0124). Other pairs of gene flow were all lower than 0.0066 (Fig. 2b), also suggesting a higher contemporary gene flow from northern to southern Xinjiang via the QGM region rather than the reverse.

### Chloroplast DNA variation and phylogenetics

The aligned cpDNA *trn*K data matrix was 2528 bp in length. A total of 25 haplotypes were identified in the 38 *P. euphratica* and *P. pruinosa* populations, based on 18 nucleotide substitutions and six indels (See Additional file 1: Table S4). Twenty haplotypes (H01–H20) occurred in *P. euphratica*, eight (H15, H19–H25) occurred in *P. pruinosa*, and three were shared by the two species (H15, H19, and H20). Another haplotype that differed from all 25 haplotypes mentioned above (by at least nine substitutions) was identified for the three *P. ilicifolia* individuals.

Haplotype diversity (*H*_d_) ranged from 0.476–0.668 and 0–0.810 within the five *P. pruinosa* populations, and 33 *P. euphratica* populations, respectively (Table 1). The level of total genetic diversity, *H*_T,_ was 0.730 and 0.705, across populations of *P. pruinosa* and *P. euphratica*, respectively. The population differentiation was larger for *P. euphratica* (*G*_ST_ = 0.391) than it was for *P. pruinosa* (*G*_ST_ = 0.183). Among the three regions of *P. euphratica*, the population from southern Xinjiang had the lowest chloroplast diversity and population divergence (Table 2).

**Fig. 3:**
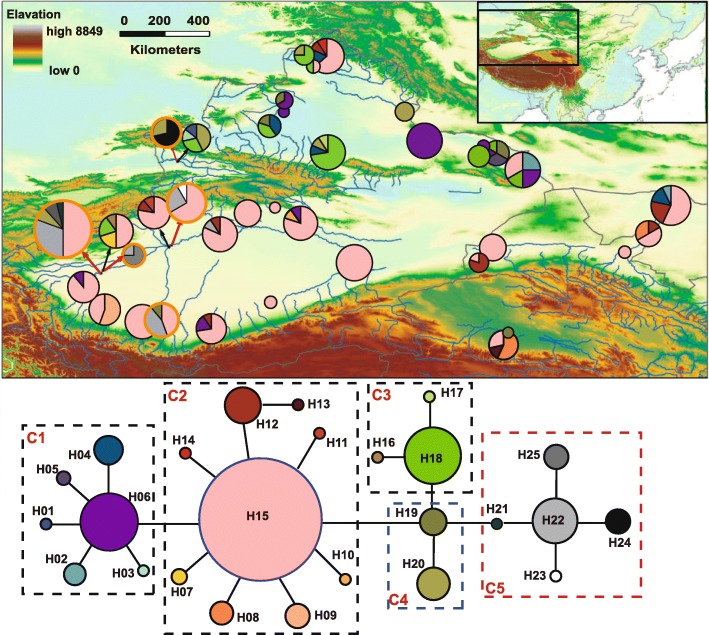
Geographical distributions and network of the cpDNA haplotypes in *Populus euphratica* (black cycle) and *P. pruinosa* (red cycle) populations. All lines joining haplotypes represent only one substitution or indels mutation, except the line between H04 and H06, which represents two substitutions. The map was created using the ArcMap package in ArcGIS ver. 9.2 (http://www.esri.com/software/arcgis)

All 25 of the cpDNA haplotypes from *P. euphratica* and *P. pruinosa* were connected into a network by one mutation between each other (except two between H04 and H06; Fig. 3), and the haplotype from *P. ilicifolia* was connected with H15 by 11 mutations (not shown). The network could be roughly classified into five clades. Clade 1 (H01–H06), Clade 2 (H07–H15), and Clade 3 (H16–H18) were composed of haplotypes that only occurred in *P. euphratica*, except H15, which was located in the center of the network and was shared by the two species and was widespread (50.5%). Clade 4 (H19–H20) was located in the center of the network and was also shared by the two species, and Clade 5 (H21–H25) was composed of haplotypes that only occurred in *P. pruinosa*. The haplotype distribution in *P euphratica* populations from northern and southern Xinjiang was clearly different. Haplotypes from Clade 1 and Clade 3 were mainly distributed in the northern Xinjiang populations, while haplotypes from Clade 2 were mainly found in southern Xinjiang populations and eastern populations. H15 that mainly occurred in southern Xinjiang and QGM regions was found in three northern Xinjiang populations: YWX, BEJa, and BEJb.

**Fig. 4:**
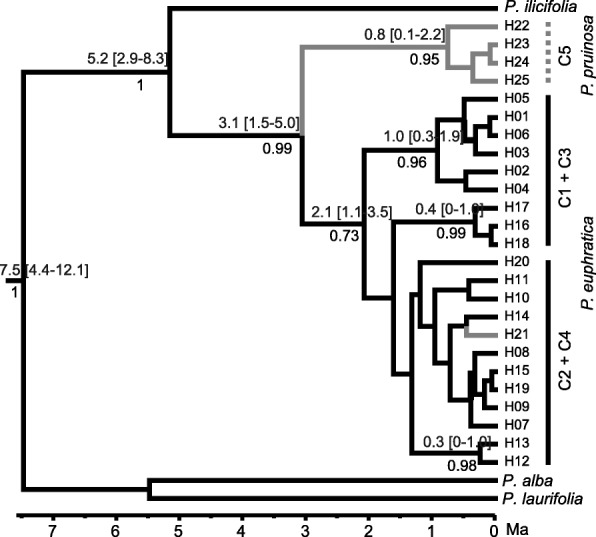
BEAST-derived chronograms of cpDNA haplotypes based on a *trn*K sequence. Numbers below branches denote posterior probabilities and those above branches indicate the divergent time [95% HPD] of the right nodes

The phylogenetic tree showed that, when *P. alba* and *P. laurifolia* were used as outgroups, all haplotypes from *P. euphratica*, *P. pruinosa*, and *P. ilicifolia* formed a monophyletic group with a support probability of 1 (Fig. 4). Within the monophyletic group, the haplotype identified in *P. ilicifolia* was divergent from all other haplotypes, with a support probability of 1, at around 5.2 Ma (95% CIs: 2.9–8.3 Ma). All other haplotypes could be roughly classified into four lineages, with a support probability of higher than 0.7. The four *P. pruinosa* private haplotypes (H22–H25), that belonged to Clade 5 of the haplotype network, formed a lineage that was divergent at around 3.1 Ma (95% CIs: 1.5–5.0 Ma). Haplotypes from northern Xinjiang formed two lineages, which corresponded well to Clade 1 and Clade 3 in the haplotype network, and were divergent at around 2.1 Ma (95% CIs: 1.0–3.5 Ma) and at around 1.5–2.0 Ma, respectively. All other haplotypes formed the fourth lineage.

### Present and past distribution of desert poplar

**Fig. 5:**
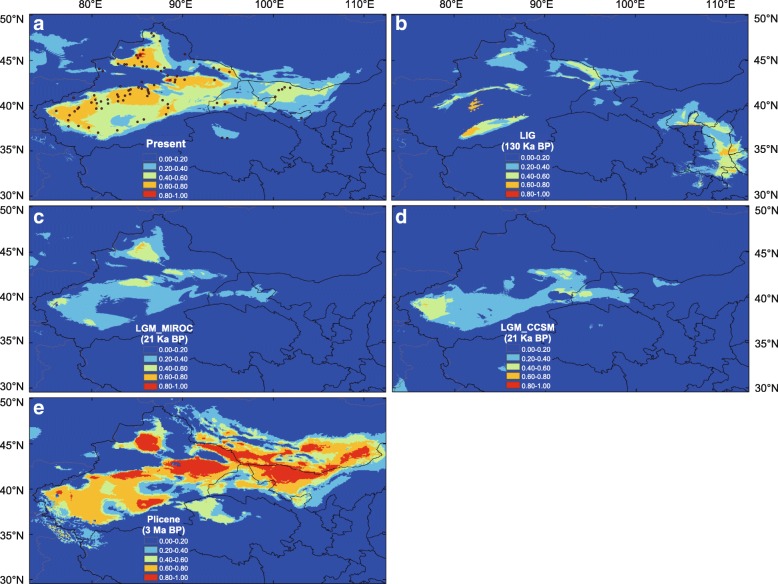
Modelled climatically suitable areas for the Euphrates poplar in China at different times: (**a**) the present; (**b**) the last interglacial (LIG: c. 130 Ka BP); (**c**) the last glacial maximum (LGM: c. 21 Ka BP) under the Model for Interdisciplinary Research on Climate (MIROC) model; (**d**) the LGM under the Community Climate System Model (CCSM), and (**e**) the Pliocene (3 Ma BP). The logistic value of habitat suitability is shown according to the color-scale bars. The map was downloaded from China’s national fundamental geographic information system

The ENM analysis based on all records of *P. euphratica* predicted completely separate potential distributions in China and those regions west of China, e.g., central Asia, Europe, and northern Africa, in all models, except for a slight connection at the western edge of the QTP (See Additional file 1: Figure S5). To specify the suitability of the estimation of the Euphrates poplar in China, we considered the ENM results based on Chinese records only. This resulted in a high predictive power, with an area under the curve of 0.941 ± 0.054. The prediction of a model over the present bioclimatic conditions showed a good to moderate suitability of the species’ extant distribution in Xinjiang province, northern Gansu province, eastern Inner Mongolia province, and a few low-suitability distributions in Qinghai province (Fig. 5a), which is consistent with the present distribution of the Euphrates poplar in China. Both the MIROC model (Fig. 5b) and the CCSM model (Fig. 5c) predicted only a few moderate suitability distributions scattered in Xinjiang province at the LGM. The prediction of the LIG also showed some moderate and scattered suitability distributions, and suitable habitats still occurred in regions further to the east (Fig. 5d). Furthermore, highly suitable habitats were predicted to be widely distributed in northwest China under the bioclimatic conditions of 3 Ma (Fig. 5e), indicating a much wider distribution range of the Euphrates poplar during the Pliocene. The ENM analysis for *P. pruinosa* also had a high predictive power, with an area under the curve of 0.966 ± 0.066. The predicted distribution range was also much wider during the Pliocene than at the present, and was slightly contracted during the LGM, but more restricted during the LIG (See Additional file 1: Figure S6).

## Discussion

### The Tianshan Mountains as a geological barrier to population divergence

Both the cpDNA *trn*K sequence and microsatellite variations demonstrate the apparent differentiation between northern and southern Xinjiang populations of Euphrates poplar, suggesting a long period of isolation between these populations. Our analysis based on nuclear microsatellites found very low levels historic and concurrent gene flow between Euphrates poplar populations from these two regions. Moreover, the limited gene flow between northern and southern Xinjiang probably occurred via the QGM region, and a higher gene flow was found from northern to southern Xinjiang via QGM than in the reverse direction (Fig. 2). Clearly, the formation of the Tianshan Mountains limited both seed dispersal and pollen flow among populations from these two regions.

Chronostratigraphic framework results from various locations within the Tianshan Mountains generally show that deformation occurred during four time intervals: early Miocene (25–20 Ma), Middle Miocene (17–15 Ma), early late Miocene (11–10 Ma), and late Miocene (7–5 Ma) [7]. Our results suggest that the chloroplast lineages distributed in northern Xinjiang became divergent from those in southern Xinjiang during the early Pleistocene, ca. 1.5–2.1 Ma (Fig. 4). Based on microsatellites and using DIYABC software, it was estimated that the divergence time between the northern and southern Xinjiang groups (t2) was 18,500 generations (95% CIs: 8100–32,800) (Table 3), which was converted to 0.37 Ma (95% CI: 0.16–0.65 Ma), assuming 20 years’ generation time. These estimated divergence times were much later than the latest Miocene deformation of the Tianshan Mountains suggested by the chronostratigraphic framework. One possible explanation for this is that the latest deformation of the Tianshan Mountains was much later than previously suggested, for example during the Pleistocene rather than the Miocene [55, 56]. However, Renner (2016) reported that the majority of phylogeny-cum-biogeography studies tended to support recent QTP uplift phases (e.g., 20–0.5 Ma). The young species divergences inferred in these studies may not be simply driven by the QTP uplift, but may reflect recent re-colonization of the plateau from possible refugia or be due to local habitat differences [57]. Thus, the later estimated divergence time in our current analysis suggested that the gene flow between Euphrates poplar populations from northern and southern Xinjiang regions had not been fully prevented by the latest Miocene deformation of the Tianshan Mountains. When Euphrates poplar was distributed widely during the Pliocene (Fig. 5e), gene flow between these two regions could still occur via the QGM region (Fig. 2).

### Influence of quaternary climatic oscillations

In northwest China, despite the absence of major Quaternary glaciations, significant climatic oscillations still occurred. During the Pleistocene glaciations, species at lower latitudes were subjected to extreme aridity as well as lower temperatures (Willis and Niklas, 2004). Sandy desert and gobi (stony desert) expansion has caused the habitat fragmentation of desert plants populations [6]. We suggest that the historical climate has greatly influenced the population demography of the Euphrates poplar in northwest China. First, the ENM estimated that suitable habitats of the Euphrates poplar in northwest China was much wider during the Pliocene (Fig. 5e), but decreased substantially during the LGM (Fig. 5b and c) and the LIG (Fig. 5d). This suggested that both the glacial (driven by EAWM) and interglacial (driven by the EASM) climates could have greatly contracted the habitat of this species and finally fragmented its distribution range (Fig. 5b, c, and d). Second, the ‘star-like’ phylogeny of haplotypes also supports a population bottleneck in the historical demography of *P. euphratica*, with the occurrence of a single, common, and often frequent ancestral haplotype (H15) in all populations of southern Xinjiang suggesting a significant historical bottleneck. Finally, the DIYABC analysis suggested that current Euphrates poplar distributions in the QGM region were created by an admixture of the northern and southern Xinjiang gene pools 336 generations ago (95% CIs: 65–1610; Scenario 5 see Additional file 1: Figure S3). Using 20 years as the generation time, an admixture of the two gene pools dates back to 6720 years ago, corresponding to the postglacial period. Thus, the current Euphrates poplar distribution in the QGM region was because of postglacial population expansion from the northern and southern Xinjiang populations. Because the gene flow between northern and southern Xinjiang mainly occurred via the QGM region (Fig. 2), the absence of the distribution in the QGM region during the glacial period would have further blocked gene flow between the populations of these two regions. Therefore, we conclude that when the deformation of the Tianshan Mountains limited the gene flow of *P. euphratica* populations from northern and southern Xinjiang, the Pleistocene climatic oscillations further accelerated the population divergence between these two regions. The cycles of the monsoonal climatic oscillations likely played a key role in the habitat fragmentation and intraspecific divergence of *P. euphratica*. The higher gene flow from northern to southern Xinjiang via the QGM than in the reverse direction (Fig. 2) indicated that the EAWM accelerated the gene flow of the desert poplar along the latitudinal direction. Similar results were found in another desert plant, *Reaumuria soongarica* [58].

The higher genetic diversity in northern Xinjiang than in southern Xinjiang in both the nuclear and chloroplast genome of *P. euphratica* (Table 2) suggested a smaller influence of historical climatic oscillations in the former region, although artificial introductions might also have contributed, e.g., population BEJa, whose chloroplast haplotypes and ancestry both belonged to southern Xinjiang according to results of Bayesian clustering analysis. The high differentiation among populations from northern Xinjiang suggested multiple historical isolations in the distribution of *P. euphratica*. The heterogeneous geology in northern Xinjiang might have facilitated the existence of multiple glacial refugia. The Ili Valley, located near the juncture between the northern and southern branches of the Tianshan Mountains, has been shown to be a glacial refugium for plants [59]. In our analysis, *P. euphratica* in Ili Valley also held distinct chloroplast haplotypes (Fig. 2), supporting the existence of a glacial distribution. The Altay-Tianshan Mountains that were found to include glacial refugia for many plants [6] may also be helpful for the glacial distribution of *P. euphratica*. Climatic oscillations have caused extreme aridity and the expansion of sandy deserts in southern Xinjiang regions [6], which would have further led to historical habitat shrinkage for *P. euphratica*.

### Genetic diversity of desert poplar and conservation applications in China

Consistent with a previous study [18], our analyses found that *P. euphratica* differed from *P. pruinosa* in both the nuclear and chloroplast genome, although there were several hybrids and a shared ancestry of cpDNA haplotypes (Figs. 1 and 3). For both species, clone diversity was low within many populations, suggesting low regeneration by seeds/seedlings of these two desert species in many locations. This was particularly true of the DH population that is located in the Euphrates poplar forest in the Dunhuang reserve, and for TLHa and TLHb, the only two high-altitude populations located in the Chaidamu Basin, with only one genet found in each of them. However, for these populations, a larger sample size and greater distance between individuals were represented during sampling. Both *P. euphratica* and *P. pruinosa* can regenerate from seed/seedlings and root suckers [20, 60], while seedlings can emerge only after flooding events [61]. Cao [60] suggested that water shortages and river channeling due to water usage and altered river flows might have resulted in no safe sites on river banks for seed germination in the National Natural Reserve of *P. euhpratica*, in Ejina. This would lead to a failure of *P. euphratica* to regenerate from seed, with root suckers being the main source of recruitment in some fields [60]. Thus, the low clonal diversity might reflect a water shortage and low groundwater table in these regions. To preserve and restore desert poplars, it is necessary to produce wet conditions favoring seed germination and establishment by regulating stream flow, for example, releasing sufficient water during peak seed rain [60].

We found that the overall gene diversity (*H*_T_) based on MLGs was similar for *P. euphratica* (0.594) and *P. pruinosa* (0.565). The genetic diversity at a population level was also comparable between the two species (*H*_O_ = 0.484 and *H*_E_ = 0.560 for *P. euphratica* and *H*_O_ = 0.538 and *H*_E_ = 0.558 for *P. pruinosa*). These values were lower than those stated in a previous study of the two species [62], possibly due to the difference in polymorphic loci selection used in the analyses. However, our genetic diversity estimates of these two desert poplars are still at an intermediate level compared with other congeneric species that have been estimated using microsatellite markers, such as *P. alba* (*H*_O_ = 0.341, *H*_E_ = 0.368) and *P. tremula* (*H*_O_ = 0.483, *H*_E_ = 0.492) [63], and *P. nigra* (*H*_O_ = 0.70, *H*_E_ = 0.73) [64]. This suggests that genetic diversity for the two endangered species in northwest China is not as low as we originally assumed.

Among *P. euphratica* from the three regions, populations from northern Xinjiang province held the highest nuclear and chloroplast genetic diversity, while populations from southern Xinjiang held the lowest genetic diversity (Table 2). The higher genetic diversity in northern Xinjiang reflects the larger effective population size and greater endemic gene resources there than in southern Xinjiang, although the latter region represents the widest Euphrates poplar distribution globally [16]. Furthermore, in northwest China, natural desert poplar reserves were mainly established in the southern Xinjiang region, e.g., Tarim national natural reserve, and in the QGM region, e.g., Ejina national natural reserve and the Dunhuang reserve. None were established in the northern Xinjiang region. Thus, we suggest that to restore the genetic resources of the Euphrates poplar, more effort is needed to protect the populations in the northern Xinjiang region.

## Conclusions

Studies in the Hengduan Mountains and adjacent regions in southwest China found that both orogeny and past climatic changes have contributed to high plant diversity [2–4]. However the roles of geology and climatic oscillations in driving the population demography and divergence of plants on the northern edge of the QTP in arid northwest China were still unknown. Our current study found that the orogeny of the Tianshan Mountains has somewhat impeded gene flow between *P. euphratica* populations from northern and southern Xinjiang; while Quaternary climatic aridification has caused significant habitat fragmentation and population contraction, which further accelerated the population divergence. These results provide a new insight into the low diversity of plants in this arid region. In addition, we also found that a water shortage and low groundwater table have resulted in a low regeneration rate of seed/seedlings in many populations of both *P. euphratica* and *P. pruinosa*. To better restore the genetic resources of the desert poplars, more effort is needed to encourage seed germination and seedling establishment, and to protect populations distributed in the northern Xinjiang region, which hold the highest genetic diversity and a large amount of endemic gene diversity.

## Additional file

Additional file 1: **Figure S1**. Sampling location of *Populus euphratica* and *P. pruinosa* populations. **Figure S2**. Frequency distribution of the pairwise distance distribution between individuals based on multilocus genotypes. **Figure S3**. The five scenarios tested in the DIYabc analysis. **Figure S4**. Inference of the most probable number of clusters (K) using STRUCTURE software. **Figure S5**. Modelled climatically suitable areas for Euphrates poplar. **Figure S6**. Modelled climatically suitable areas for *P. pruinosa* **Table S1**. Description of *Populus pruinosa* and *P. euphratica* populations analysed. **Table S2**. Description and references of the 17 microsatellite loci analysed for this study. **Table S3**. Diversity and differentiation for the 17 microsatellite loci analysed in *Populus euphratica* and *P. pruinosa*. **Table S4**. Variable sites of the aligned sequences of chloroplast DNA fragments in 25 haplotypes of *Populus euphratic* and *P. pruinosa* in northwest China. (DOCX 2025 kb)

## Abbreviations

CCSM: Community Climate System Model
CIs: Confidence intervals
cpDNA: Chloroplast DNA
EASM: the East Asian summer monsoons
EAWM: the East Asian winter monsoons
ENM: Ecological Niche Modeling
LGM: Last glacial maximum
LIG: Last interglacial
MIROC: the Model for Interdisciplinary Research on Climate
ML: Maximum likelihood
MLGs: Multilocus genotypes
PCR: Polymerase chain reaction
PP: Posterior probabilities
QGM: Qinghai province, Gansu province, and Inner Mongolia province
QTP: Qinghai-Tibetan Plateau
