# Supplementary material for: Phylogeographic patterns of the desert poplar in Northwest China shaped by both geology and climatic oscillations
Source: BMC Evol Biol. 2018 May 25;18:75. doi: 10.1186/s12862-018-1194-1 (PMC5970483; doi:10.1186/s12862-018-1194-1)
Supplement: Supplementary file 1 — Figure S1. Sampling location of Populus euphratica and P. pruinosa populations. Figure S2. Frequency distribution of the pairwise distance distribution between individuals based on multilocus genotypes. Figure S3. The five scenarios tested in the DIYabc analysis. Figure S4. Inference of the most probable number of clusters (K) using STRUCTURE software. Figure S5. Modelled climatically suitable areas for Euphrates poplar. Figure S6. Modelled climatically suitable areas for P. pruinosa Table S1. Description of Populus pruinosa and P. euphratica populations analysed. Table S2. Description and references of the 17 microsatellite loci analysed for this study. Table S3. Diversity and differentiation for the 17 microsatellite loci analysed in Populus euphratica and P. pruinosa. Table S4. Variable sites of the aligned sequences of chloroplast DNA fragments in 25 haplotypes of Populus euphratic and P. pruinosa in northwest China. (DOCX 2025 kb) [file 12862_2018_1194_MOESM1_ESM.docx]

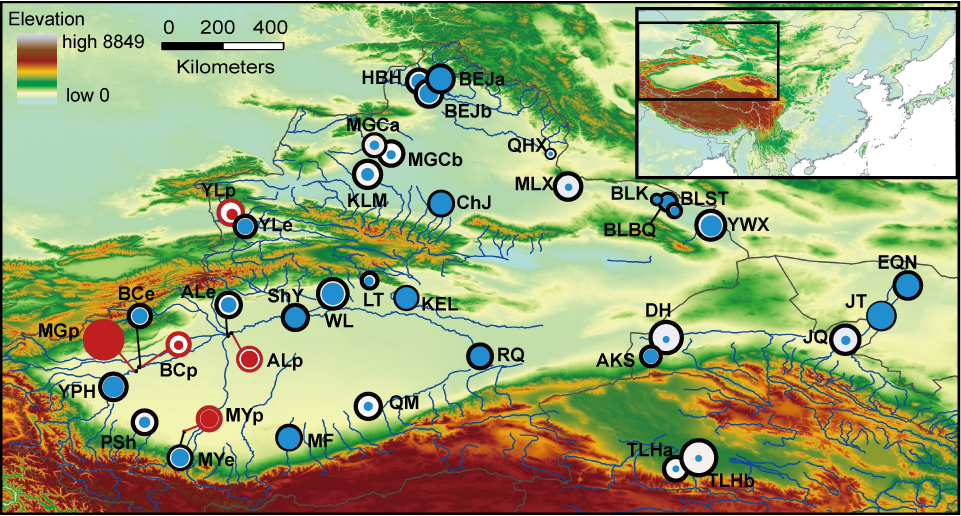


Fig. S1 Sampling location of *Populus euphratica* and *P. pruinosa* populations. The size of outer black or red circles represents the relative sample size for each location. The blue or red filled areas within the each black circle represents the number of multilocus genotypes (MLGs) in each *P. euphratica* and *P. pruinosa* population, respectively. The map was created using the ArcMap package in ArcGIS ver. 9.2 (http://www.esri.com/software/arcgis).


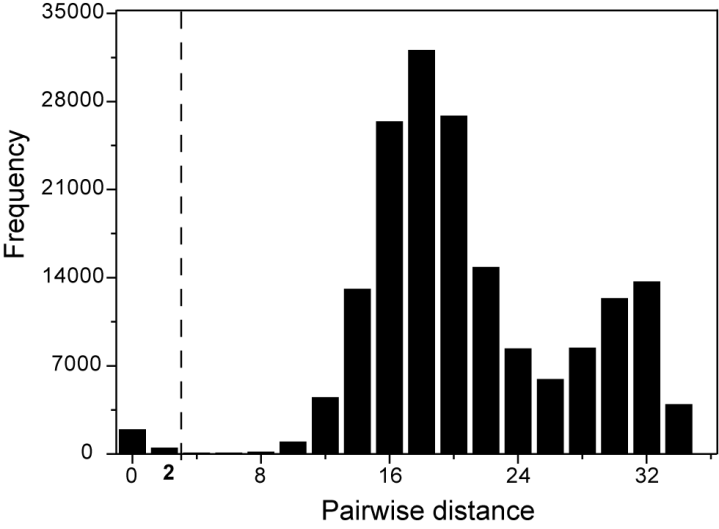


Fig. S2. Frequency distribution of the pairwise distance distribution between individuals based on multi-locus genotypes. A threshold 2 was used to identify ramets belonging to the same genet.


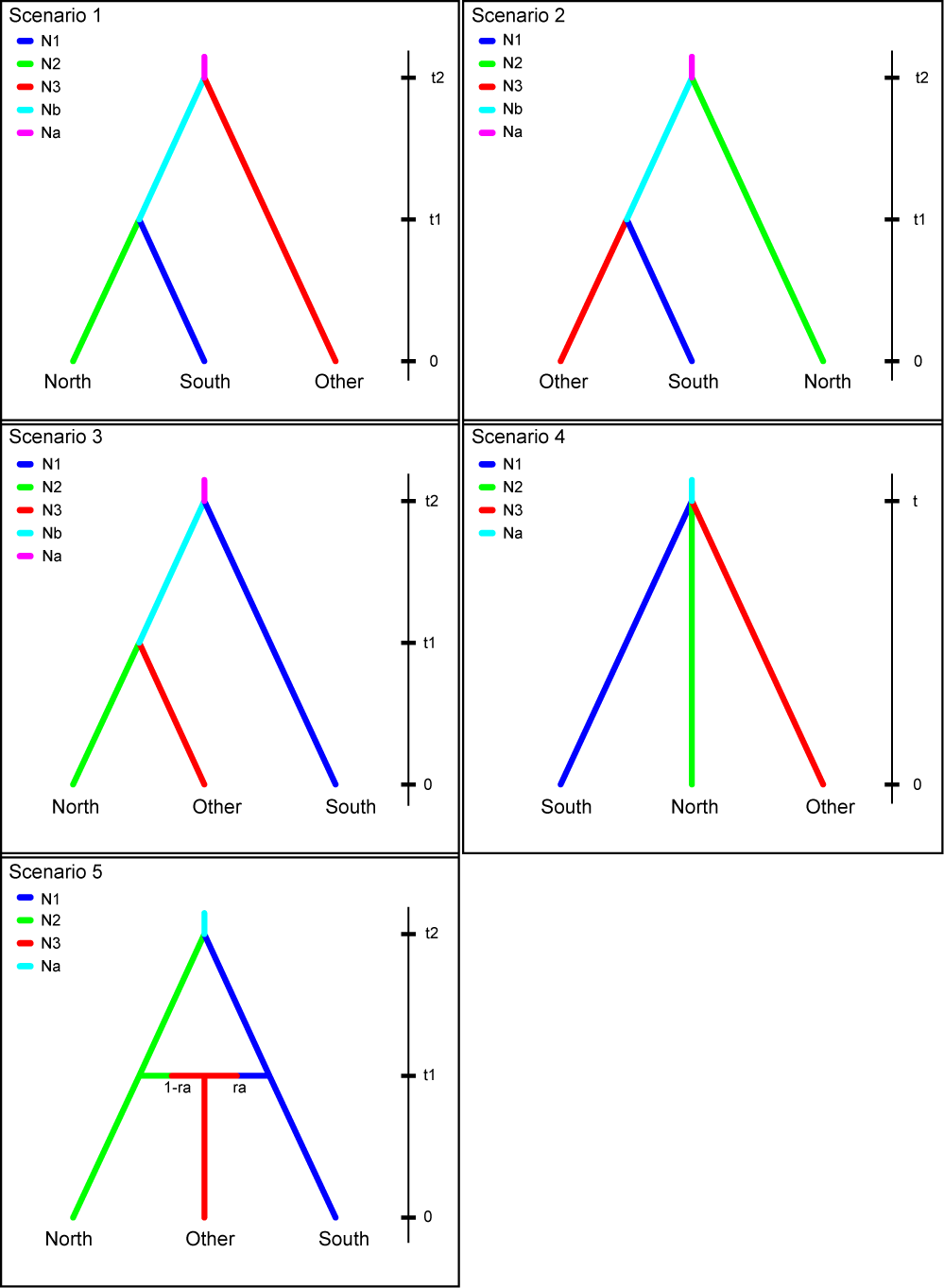


Fig. S3. The five scenarios tested in the DIYabc analysis. North, Euphrates populations from Northern Xinjiang; South, Euphrates populations from Southern Xinjiang; Other, Euphrates populations from Qinghai, Gansu and Inner Mongolia provinces.


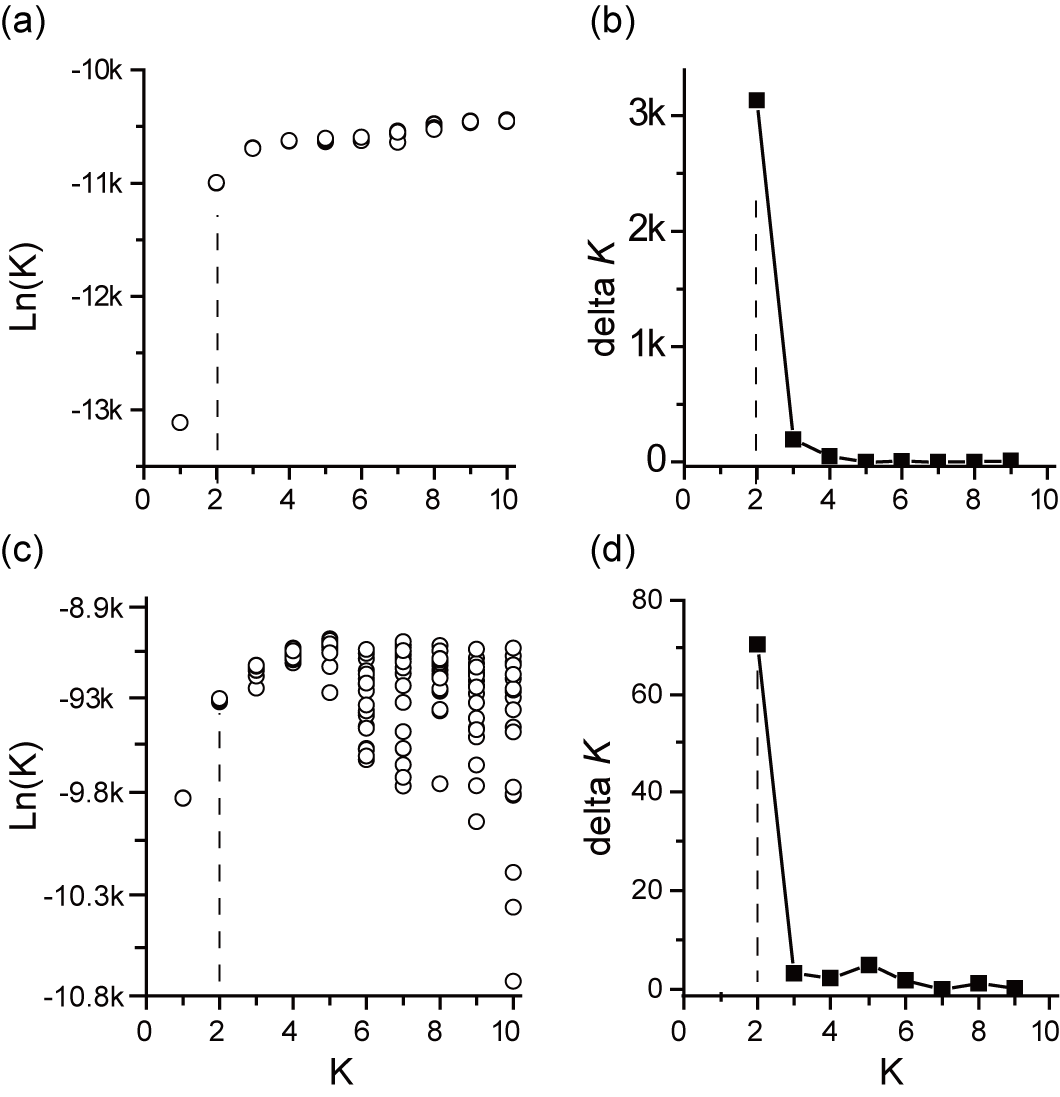


Fig. S4. Inference of the most probable number of clusters (K) using STRUCTURE software. (a) Log-likelihood value of data, Ln P(K), as a function of K for all sampled multi-locus genotypes of *P. euphratica* and *P. pruinosa*. (b) Second-order change of the log-likelihood of the data (Delta K) as a function of K for all sampled multi-locus genotypes of *P. euphratica* and *P. pruinosa*. (c) Log-likelihood value of data, Ln P(K), as a function of K for all sampled multi-locus genotypes of *P. euphratica;* (b) Second-order change of the log-likelihood of the data (Delta K) as a function of K for all sampled multi-locus genotypes of *P. euphratica*. Analysis was done based on 17 nuclear microsatellite loci, and calculated over twenty replicates.


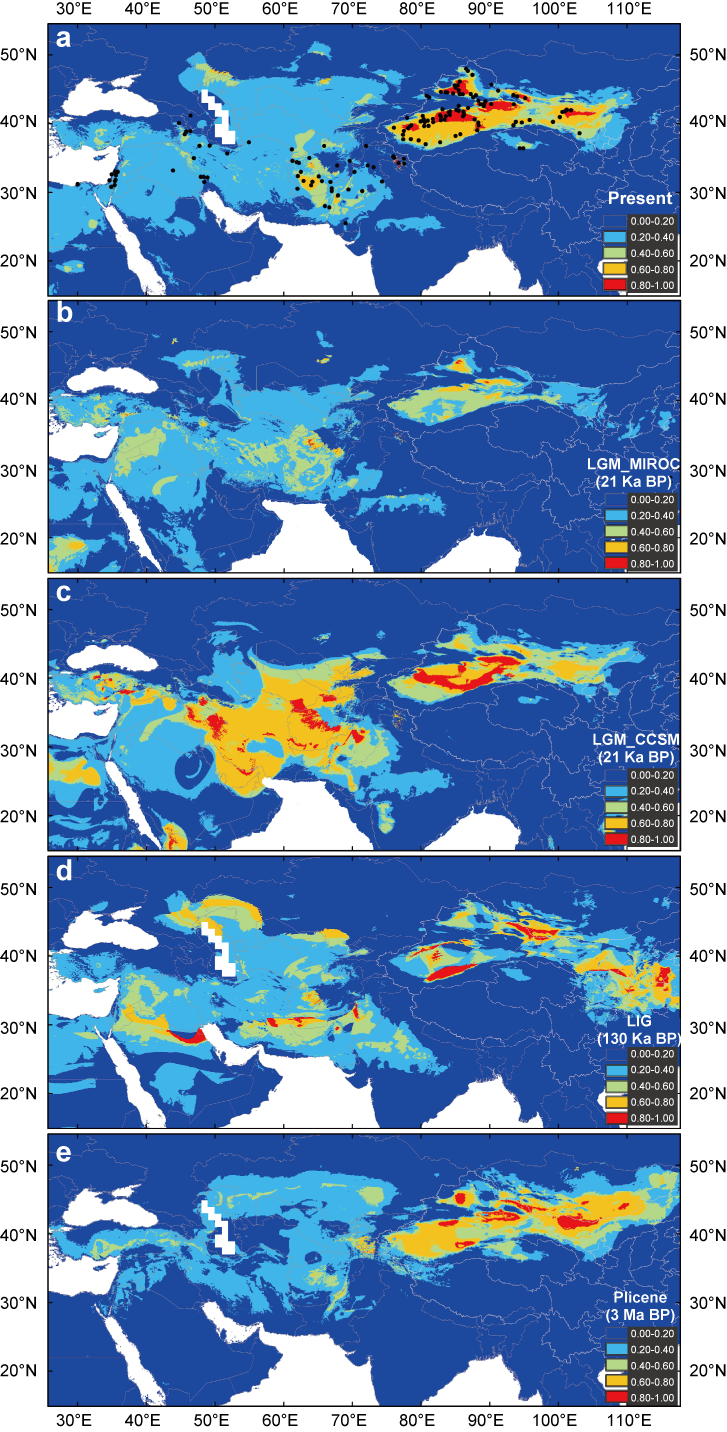


Fig. S5. Modelled climatically suitable areas for Euphrates poplar at present (a), the last interglacial (LIG: c. 130 Ka BP; b), the last glacial maximum (LGM: c. 21 Ka BP) under the MIROC model (c), the LGM under the CCSM model (d) and the Pliocene (3 Ma BP; e). The logistic value of habitat suitability is shown according to the colour-scale bars. The map was downloaded from China's national fundamental geographic information system.


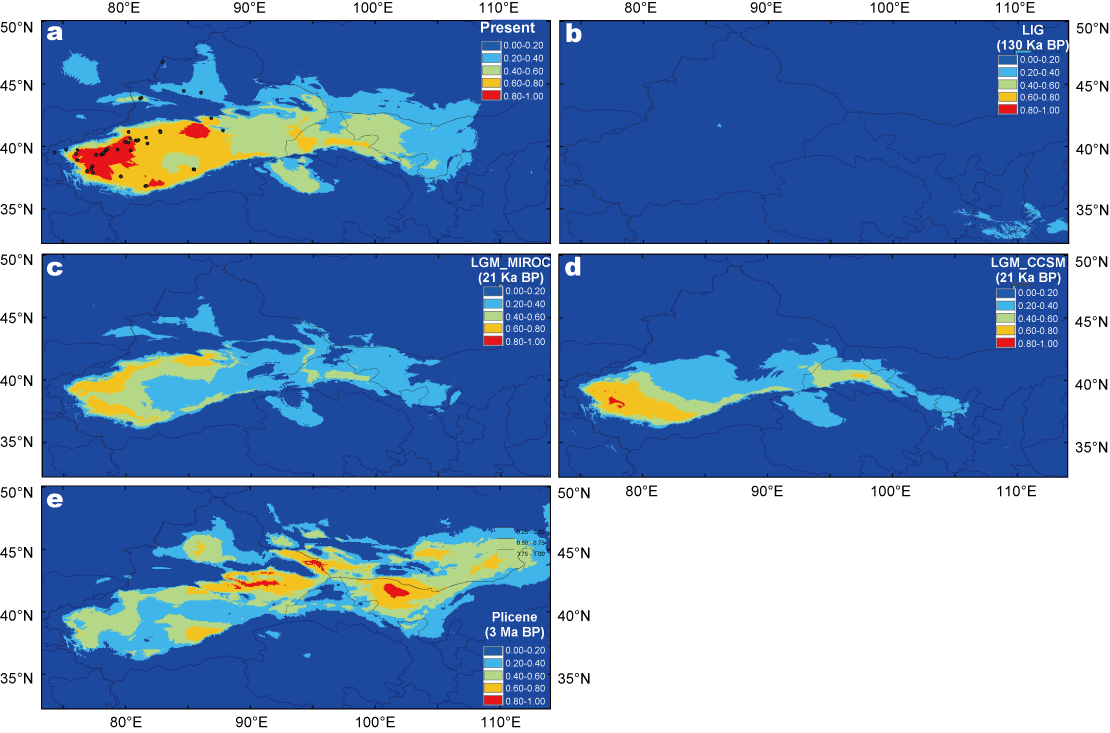


Fig. S6. Modelled climatically suitable areas for *P. pruinosa* at present (a), the last interglacial (LIG: c. 130 Ka BP; b), the last glacial maximum (LGM: c. 21 Ka BP) under the MIROC model (c), the LGM under the CCSM model (d) and the Pliocene (3 Ma BP; e). The logistic value of habitat suitability is shown according to the colour-scale bars. The map was downloaded from China's national fundamental geographic information system.

Table S1. Description of *Populus pruinosa* and *P. euphratica* populations analysed.

| Species | Region/  province | Localities | Code | N | Latitude  (N) | Longitude (E) | Altitude (m) |
| --- | --- | --- | --- | --- | --- | --- | --- |
| *P. pruinosa* | Southern Xinjiang | Moyu | MYp | 15 | 37°34′48" | 79°38′11" | 1292 |
|  |  | Bachu | BCp | 14 | 39°29′50" | 78°17′26" | 1136 |
|  |  | A’laershi | Alp | 16 | 40°30′37" | 81°02′03" | 1012 |
|  |  | Maigaiti | MGp | 40 | 39°22′10" | 78°11′51" | － |
|  | Northern Xinjiang | Yili | YLp | 17 | 43°53′12" | 81°17′59" | － |
| *P. euphratica* | Southern Xinjiang | Moyu | MYe | 16 | 37°34′48" | 79°38′11" | 1292 |
|  |  | Bachu | BCe | 14 | 39°29′50" | 78°17′26" | 1136 |
|  |  | A’laershi | Ale | 17 | 40°30′37" | 81°02′03" | 1012 |
|  |  | Minfeng | MF | 16 | 37°15′51" | 82°46′48" | 1362 |
|  |  | Pishan | PSh | 16 | 37°44′32" | 78°30′27" | 1306 |
|  |  | Qiemo | QM | 18 | 38°11′29" | 85°01′09" | 1232 |
|  |  | Yuepuhu | YPH | 20 | 38°55′58" | 77°30′50" | － |
|  |  | Ruoqiang | RQ | 16 | 39°44′45" | 88°24′04" | 810 |
|  |  | Shaya | ShY | 18^*^ | 40°58′22" | 82°53′58" | 959 |
|  |  | Kuerle | KEL | 14 | 41°31′45" | 86°13′02" | 892 |
|  |  | Weili | WL | 25 | 41°39′47" | 84°02′50" | － |
|  |  | Luntai | LT | 7 | 41°59′24" | 85°08′05" | 967 |
|  | Northern Xinjiang | Yili | YLe | 13 | 43°53′12" | 81°17′59" | － |
|  |  | Changji | ChJ | 16 | 44°18′22" | 87°15′17" | 451 |
|  |  | Kelamayi | KLM | 21 | 45°14′52" | 85°03′25" | 288 |
|  |  | Moguicheng | MGCa | 15 | 46°08′04" | 85°38′50" | 303 |
|  |  | Moguicheng | MGCb | 17 | 45°58′36" | 85°35′14" | 305 |
|  |  | Buerjin | BEJa | 20 | 47°48′19" | 86°51′35" | 478 |
|  |  | Buerjin | BEJb | 20 | 47°42′18" | 86°48′26" | 479 |
|  |  | Habahe | HBH | 16 | 47°59′09" | 86°33′41" | 49 |
|  |  | Balikun | BLK | 3 | 44°10′09" | 93°25′59" | 1140 |
|  |  | Balikun | BLST | 5 | 44°00′24" | 93°51′34" | 1019 |
|  |  | Balikun | BLBQ | 9 | 43°58′25" | 94°01′15" | 586 |
|  |  | Qinghe | QHX | 3 | 45°40′49" | 90°15′21" | － |
|  |  | Mulei | MLX | 20 | 44°48′22" | 91°14′30" | 734 |
|  |  | Yiwu | YWX | 24 | 43°42′43" | 95°09′21" | 451 |
|  | Qinghai | Geermu | TLHa | 16 | 36°25′83" | 94°22′24" | 2787 |
|  |  | Geermu | TLHb | 35 | 36°28′08" | 94°21′51" | 2776 |
|  | Gansu | Akesai | AKS | 11 | 39°46′44" | 93°26′11" | 1530 |
|  |  | Jiuquan | JQ | 23 | 40°14′88" | 99°11′61" | 1190 |
|  |  | Dunhuang | DH | 28 | 40°16′51" | 93°48′22" | 1035 |
|  |  | Jinta | JT | 20 | 40°58′14" | 100°12′54" | 1065 |
|  | Inner Mongolia | Ejina | EQN | 20 | 41°57′49" | 101°04′34" | － |

^*^one individual was removed from this analysis, as a structure assignment analysis suggested it was *P. pruinosa* with a very high probability.

Table S2 Description and references of the 17 microsatellite loci analysed for this study

| Locus name | Motif | Size range (bp) | N | Reference |
| --- | --- | --- | --- | --- |
| GCPM_1011^*^ | at | 199-237 | 18 | IPGC^a^ |
| GCPM_1048 | ga | 205-229 | 11 | IPGC |
| GCPM_1065 | ac | 166-169 | 2 | IPGC |
| GCPM_1158^*^ | ctg | 236-281 | 14 | IPGC |
| GCPM_1414 | tct | 112-115 | 2 | IPGC |
| GCPM_1589 | gaa | 229-250 | 5 | IPGC |
| GCPM_1608^*^ | tg | 185-251 | 28 | IPGC |
| GCPM_162 | ctt | 220-238 | 6 | IPGC |
| GCPM_1623^*^ | at | 172-212 | 20 | IPGC |
| GCPM_1941^*^ | ttc | 152-197 | 11 | IPGC |
| GCPM_2126 | ag | 170-212 | 16 | IPGC |
| Pe2^*^ | (ct)_9_(ca)_11_ | 113-183 | 28 | ([Wu *et al.* 2008](#_ENREF_1)) |
| Pe4 | (tc)_11_ | 108-134 | 13 | ([Wu *et al.* 2008](#_ENREF_1)) |
| Pe5 | (tc)_14_ | 169-203 | 15 | ([Wu *et al.* 2008](#_ENREF_1)) |
| Pe7^*^ | (tg)_10_ | 184-216 | 14 | ([Wu *et al.* 2008](#_ENREF_1)) |
| Pe13 | (ct)_6_-(gt)_5_ | 148-152 | 3 | ([Wu *et al.* 2008](#_ENREF_1)) |
| Pe16^*^ | (ac)_17_ | 244-278 | 15 | ([Wu *et al.* 2008](#_ENREF_1)) |

^a^ International *Populus* Genome Consortium

Table S3 Diversity and differentiation for the 17 microsatellite loci analysed in *Populus euphratica* and *P. pruinosa*.

|  | *P. euphratica* | | | | | | *P. pruinosa* | | | | | |
| --- | --- | --- | --- | --- | --- | --- | --- | --- | --- | --- | --- | --- |
| LocName | *N*_A_ | *H*_O_ | *H*_S_ | *H*_T_ | *F*_ST_ | *R*_ST_ | *N*_A_ | *H*_O_ | *H*_S_ | *H*_T_ | *F*_ST_ | *R*_ST_ |
| GCPM_1011 | 18 | 0.843 | 0.842 | 0.885 | 0.069 | 0.138 | 10 | 0.617 | 0.774 | 0.769 | -0.016 | -0.066 |
| GCPM_1048 | 7 | 0.418 | 0.585 | 0.629 | 0.057 | 0.012 | 8 | 0.675 | 0.691 | 0.718 | 0.038 | 0.138 |
| GCPM_1065 | 2 | 0.060 | 0.061 | 0.064 | 0.055 | 0.055 | 2 | 0.364 | 0.401 | 0.373 | -0.031 | -0.031 |
| GCPM_1158 | 14 | 0.741 | 0.698 | 0.750 | 0.084 | 0.003 | 7 | 0.451 | 0.546 | 0.518 | -0.018 | -0.009 |
| GCPM_1414 | 2 | 0.035 | 0.033 | 0.034 | 0.077 | 0.077 | 2 | 0.123 | 0.213 | 0.205 | 0.005 | 0.005 |
| GCPM_1589 | 4 | 0.082 | 0.080 | 0.085 | 0.032 | 0.073 | 4 | 0.160 | 0.208 | 0.201 | -0.023 | -0.048 |
| GCPM_1608 | 27 | 0.846 | 0.846 | 0.908 | 0.062 | 0.283 | 20 | 0.923 | 0.813 | 0.834 | 0.014 | 0.013 |
| GCPM_162 | 6 | 0.591 | 0.511 | 0.536 | 0.054 | 0.052 | 4 | 0.573 | 0.707 | 0.696 | -0.012 | -0.029 |
| GCPM_1623 | 19 | 0.725 | 0.736 | 0.790 | 0.052 | 0.041 | 19 | 0.828 | 0.897 | 0.905 | 0.011 | 0.035 |
| GCPM_1941 | 10 | 0.544 | 0.630 | 0.637 | 0.020 | 0.047 | 9 | 0.627 | 0.639 | 0.614 | -0.021 | -0.035 |
| GCPM_2126 | 15 | 0.640 | 0.615 | 0.678 | 0.088 | -0.004 | 12 | 0.586 | 0.698 | 0.754 | 0.051 | 0.022 |
| Pe2 | 25 | 0.863 | 0.857 | 0.924 | 0.064 | 0.012 | 16 | 0.661 | 0.773 | 0.762 | -0.007 | 0.011 |
| Pe4 | 10 | 0.461 | 0.679 | 0.699 | 0.037 | 0.043 | 11 | 0.248 | 0.8 | 0.857 | 0.031 | 0.352 |
| Pe5 | 14 | 0.685 | 0.673 | 0.703 | 0.046 | 0.076 | 13 | 0.816 | 0.818 | 0.856 | 0.042 | -0.024 |
| Pe7 | 15 | 0.810 | 0.774 | 0.843 | 0.087 | 0.093 | 5 | 0.042 | 0.045 | 0.043 | -0.015 | 0.006 |
| Pe13 | 2 | 0.064 | 0.063 | 0.062 | 0.013 | 0.013 | 3 | 0.345 | 0.314 | 0.318 | 0.011 | 0.016 |
| Pe16 | 17 | 0.742 | 0.796 | 0.860 | 0.079 | 0.076 | 7 | 0.190 | 0.18 | 0.182 | 0.013 | 0.000 |
| Mean | 12.2 | 0.538 | 0.558 | 0.594 | 0.062^**^ | 0.101 | 8.9 | 0.484 | 0.560 | 0.565 | 0.009 | 0.007 |

Table S4 Variable sites of the aligned sequences of chloroplast DNA fragments in 25 haplotypes of *Populus euphratic* and *P. pruinosa* in northwest China.

| Haplotype | *trn*K variable position | | | | | | | | | | | | | | | | | | | | | | | | |
| --- | --- | --- | --- | --- | --- | --- | --- | --- | --- | --- | --- | --- | --- | --- | --- | --- | --- | --- | --- | --- | --- | --- | --- | --- | --- |
|  | 85 | 87 | 120 | 208 | 214 | 304 | 603 | 616 | 999 | 1462 | 1516 | 1548 | 1588 | 1650 | 1821 | 1848 | 1863 | 1889 | 1936 | 2049 | 2147 | 2186 | 2226 | 2360 |  |
| H01 | A | † | C | C | C | G | C | C | C | C | G | T | T | C | G | - | - | A | T | # | C | C | - | - |  |
| H02 | A | † | C | C | C | G | C | T | C | C | G | T | T | C | G | - | - | A | T | # | C | T | - | - |  |
| H03 | A | † | C | C | C | G | C | C | C | C | G | T | T | C | G | - | - | A | T | # | C | T | - | © |  |
| H04 | A | † | C | C | C | G | C | C | C | C | G | T | G | C | A | - | - | A | T | # | C | T | - | - |  |
| H05 | A | † | C | T | C | G | C | C | C | C | G | T | T | C | G | - | - | A | T | # | C | T | - | - |  |
| H06 | A | † | C | C | C | G | C | C | C | C | G | T | T | C | G | - | - | A | T | # | C | T | - | - |  |
| H07 | A | † | C | C | C | G | C | C | C | C | G | T | T | C | G | ‡ | - | G | T | # | C | T | - | - |  |
| H08 | A | † | C | C | C | G | C | C | C | C | G | T | T | C | G | - | - | G | T | # | C | T | T | - |  |
| H09 | A | - | C | C | C | G | C | C | C | C | G | T | T | C | G | - | - | G | T | # | C | T | - | - |  |
| H10 | C | † | C | C | C | G | C | C | C | C | G | T | T | C | G | - | - | G | T | # | C | T | - | - |  |
| H11 | A | † | C | C | C | G | C | C | C | C | G | T | T | C | G | - | - | G | C | # | C | T | - | - |  |
| H12 | A | † | C | C | G | G | C | C | C | C | G | T | T | C | G | - | - | G | T | # | C | T | - | - |  |
| H13 | A | † | C | C | G | G | C | C | C | T | G | T | T | C | G | - | - | G | T | # | C | T | - | - |  |
| H14 | A | † | C | C | C | G | T | C | C | C | G | T | T | C | G | - | - | G | T | # | C | T | - | - |  |
| H15 | A | † | C | C | C | G | C | C | C | C | G | T | T | C | G | - | - | G | T | # | C | T | - | - |  |
| H16 | A | † | C | C | C | G | C | C | C | C | C | T | T | C | G | - | - | G | T | # | C | T | - | - |  |
| H17 | A | † | C | C | C | G | C | C | G | C | C | T | T | C | G | - | - | G | T | - | C | T | - | - |  |
| H18 | A | † | C | C | C | G | C | C | C | C | C | T | T | C | G | - | - | G | T | - | C | T | - | - |  |
| H19 | A | † | C | C | C | G | C | C | C | C | G | T | T | C | G | - | - | G | T | - | C | T | - | - |  |
| H20 | A | † | C | C | C | G | C | C | C | C | G | T | T | T | G | - | - | G | T | - | C | T | - | - |  |
| H21 | A | † | C | C | C | G | C | C | C | C | G | T | T | C | G | - | - | G | T | - | T | T | - | - |  |
| H22 | A | † | C | C | C | T | C | C | C | C | G | T | T | C | G | - | - | G | T | - | T | T | - | - |  |
| H23 | A | † | T | C | C | T | C | C | C | C | G | T | T | C | G | - | - | G | T | - | T | T | - | - |  |
| H24 | A | † | C | C | C | T | C | C | C | C | G | T | T | C | G | - | * | G | T | - | T | T | - | - |  |
| H25 | A | † | C | C | C | T | C | C | C | C | G | C | T | C | G | - | - | G | T | - | T | T | - | - |  |

† indicates ‘ACGGAA’; ‡ indicates ‘TATAAT’; * indicates ‘TAAAAAGA ’; # indicates ‘TTTATCAA’; © indicates ‘CAAGAATTCAAA’. – indicates gap. Sequences are numbered from the 5’ to the 3’ end.
